# Transcriptomic and Metabolomic Analyses Reveal the Role of Phenylalanine Metabolism in the Maize Response to Stalk Rot Caused by *Fusarium proliferatum*

**DOI:** 10.3390/ijms25031492

**Published:** 2024-01-25

**Authors:** Jianjun Sun, Yanzhao Wang, Xingrui Zhang, Zeqiang Cheng, Yinghui Song, Huimin Li, Na Wang, Shen Liu, Zijia Cao, Hongxia Li, Wanying Zheng, Canxing Duan, Yanyong Cao

**Affiliations:** 1 Institute of Cereal Crops, Henan Academy of Agricultural Sciences, Zhengzhou 450002, China; 2 Key Laboratory of Grain Crop Genetic Resources Evaluation and Utilization, Institute of Crop Sciences, Chinese Academy of Agricultural Sciences, Beijing 100081, China; 3 The Shennong Laboratory, Zhengzhou 450002, China

**Keywords:** transcriptome, metabolome, maize stalk rot, *Fusarium proliferatum*, phenylalanine metabolism

## Abstract

Stalk rot is a prevalent disease of maize (*Zea mays* L.) that severely affects maize yield and quality worldwide. The ascomycete fungus *Fusarium* spp. is the most common pathogen of maize stalk rot. At present, the molecular mechanism of *Fusarium proliferation* during the maize stalk infection that causes maize stalk rot has rarely been reported. In this study, we investigated the response of maize to *F. proliferatum* infestation by analyzing the phenotypic, transcriptomic, and metabolomic data of inbred lines ZC17 (resistant) and CH72 (susceptible) with different levels of resistance to stalk rot. Physiological and phenotypic results showed that the infection CH72 was significantly more severe than ZC17 after inoculation. Transcriptome analysis showed that after inoculation, the number of differentially expressed genes (DEGs) was higher in CH72 than in ZC17. Nearly half of these DEGs showed the same expression trend in the two inbred lines. Functional annotation and enrichment analyses indicated that the major pathways enriched for DEGs and DEMs included the biosynthesis of plant secondary metabolites, phenylalanine metabolism, biosynthesis of plant hormones, and plant–pathogen interactions. The comprehensive analysis of transcriptome and metabolome data indicated that phenylalanine metabolism and the phenylalanine, tyrosine, and tryptophan biosynthesis pathways played a crucial role in maize resistance to *F. proliferatum* infection. In addition, a transcription factor (TF) analysis of the DEGs showed that several TF families, including MYB, bHLH, NAC, and WRKY, were significantly activated after inoculation, suggesting that these TFs play important roles in the molecular regulatory network of maize disease resistance. The findings of this study provide valuable insights into the molecular basis of the response of maize to *Fusarium proliferatum* infection and highlight the importance of combining multiple approaches, such as phenotyping, transcriptomics, and metabolomics, to gain a comprehensive understanding of plant–pathogen interactions.

## 1. Introduction

Maize (*Zea mays* L.) is an important food and feed crop, industrial raw material, and energy plant. Corn, in the world’s sown surface area, is second only to wheat and rice, ranked third, but the total yield of corn ranks first and it is truly the world’s foremost food crop, while also being China’s largest food crop. Chinese corn production in the world ranks second only to that of the United States [1]. China is both a corn-producing country and a corn-consuming country, so corn is of great significance to China’s food security. However, the frequent and high incidence of pests and diseases has caused great potential risks to the safety of that food production. Like other crops, maize is susceptible to a variety of diseases during growth, with stalk rot being one of the diseases that seriously affects maize yield and quality [2]. Some studies have reported that stalk rot usually leads to a 10% reduction in maize yields [3], with yield losses of up to 30–50% in areas of high incidence [4]. In addition to yield losses, stalk rot can also cause lodging, which further reduces yields, affects kernel quality, and is detrimental to mechanized harvesting [5,6].

Maize stalk rot is a common soil-borne disease in the field, and the causal organisms are complex; it can be caused by a variety of Fusarium or Pythium fungi, either alone or in a complex infestation. The pathogen of maize stalk rot in northern China is mainly Fusarium, with *Fusarium graminearum*, *Fusarium proliferatum*, and *Fusarium verticillioides* being the most severely damaging [7,8,9]. Previous research on maize stalk rot pathogens has mainly focused on *F. graminearum*, but, in recent years, it was found that *F. proliferatum* and *F. verticillioides* have become the dominant pathogens [10,11]. In recent years, with climate change, straw return to the field, the promotion of no-tillage cultivation methods, and variety changes, field pathogens continue to accumulate, resulting in maize stalk rot in China and aggravating its trend of occurrence [12,13].

While some progress has been made in agronomic and chemical practices to prevent maize stalk rot pathogens [14], to date, no specific resistance genes have been identified that are immune to Fusarium stalk rot, and resistance in commercial hybrids has been lower than expected [15,16]. However, the incidence of the disease in the field survey shows that there were significant differences in the resistance of different maize varieties or germplasm resources to stalk rot, and resistant varieties can significantly reduce the incidence of the disease and reduce the loss of yield [17]. Therefore, analyzing the pathogenesis of stalk rot, mining resistance genes, and breeding resistant varieties are the most economical and effective measures to prevent stalk rot. Several studies have reported the key genes and potential mechanisms of stem rot caused by *F. graminearum* and *F. verticillioides* [18,19], however, research on stem rot caused by *F. proliferatum* is still limited, which has greatly restricted the progress of the effective control of this disease.

Stalk rot frequently occurs in the middle to late stages of corn growth, and *F. proliferatum* is capable of causing the blackening and rotting of plant stems, the degradation of cell walls, and the production of toxic substances such as fumonisins (FBs) [20]. In response to infestation by various types of pathogens, plants have evolved many complex mechanisms to respond to these adverse factors. Plants can recognize pathogen-associated molecular patterns (PAMPs) through pattern recognition receptors on their cells’ surfaces and sense pathogen effectors through resistance proteins, producing PTI (PAMP-triggered immunity) and ETI (effector-triggered immunity) [21]. In addition, plants defend themselves against pathogenic microbial attacks through a variety of defense responses, such as regulating the expression of key metabolic pathway genes while activating the expression of genes directly involved in defense [22], initiating hormone signaling regulation and transmission [23], releasing reactive oxygen species (ROS) to induce the expression of stress proteins, and regulating relevant disease resistance genes through transcription factors.

Many secondary metabolites play an essential role in plant disease tolerance, among which the phenylalanine metabolic pathway is not only involved in the synthesis of the secondary metabolites lignin, flavonoids, and phenolic substances, but also in the synthesis of an important hormone signaling molecule, salicylic acid (SA), which is closely related to plant disease resistance [24].

This pathway involves the formation of various pathogen-resistant substances catalyzed by a series of enzymes, such as PPO, that oxidize phenolics while producing toxic quinine, which can effectively inhibit the invasion of fungal pathogens [25]. Lignin is an important component of the plant cell wall and is a monolignin polymer from the phenylpropanoid pathway. It also serves as a barrier against the growth and multiplication of pathogenic organisms and is an important defense against pathogen invasion and expansion. Numerous studies have shown that phytohormones, especially SA and JA, play an important role in plant resistance to pathogen infection [26]. The involvement of phytohormone signaling in disease resistance is inextricably linked to the regulation of transcription factors, which play an important role as regulatory genes in the plant response to pathogens [27]. It has been reported that transcription factors of the MYB, NAC, and WRKY families are involved in the JA signaling pathway, and these transcription factors and their homologs may bind to JAZs and specifically regulate the JA response [28]. In addition, NAC can also be induced to be expressed upon pathogen infection and play a role in plant resistance to necrotrophic pathogens [29].

In this study, we analyzed the differences in gene expression and metabolite biosynthesis in the transcriptome and metabolome using the maize inbred lines ZC17 and CH72, which have different resistances to *F. proliferatum*. We found that the phenylalanine metabolism pathway played a key role in enhancing the response of maize to *F. proliferatum* infestation. These results provide a theoretical basis for the future breeding of maize varieties with enhanced stalk rot resistance.

## 2. Results

### 2.1. Phenotypic and Physiological Characteristics of the Inbred Lines ZC17 and CH72 with Different Levels of Resistance to F. proliferatum after Inoculation

**Figure 1:**
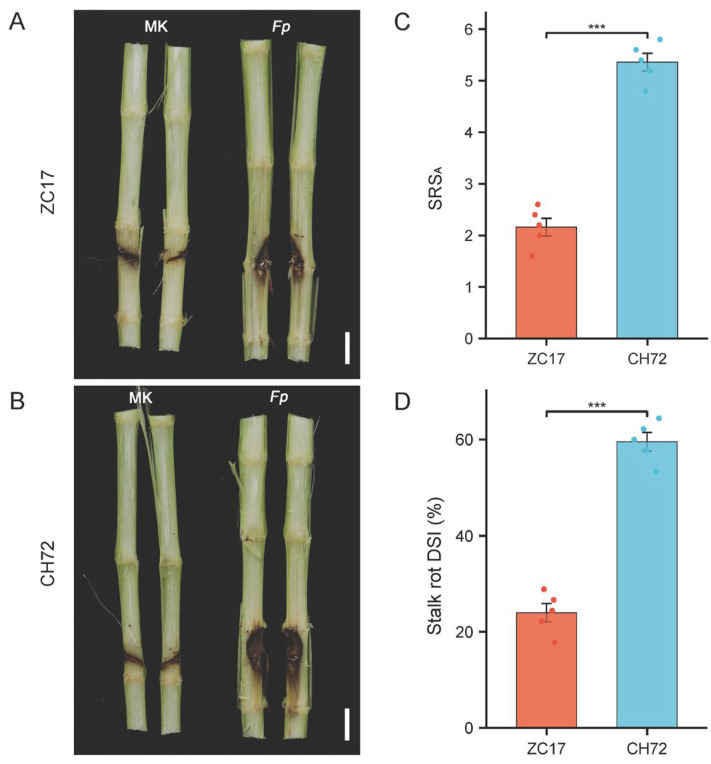
Phenotypes and disease symptoms of different resistant maize inbred lines after inoculation. Characterization of stem disease incidence of resistant inbred line ZC17 (**A**) and susceptible inbred line CH72 (**B**) in *F. proliferatum*-inoculated and mock-inoculated groups at 5 days post inoculation. Scale bars next to CH72 and ZC17 are 2 cm. Statistical analysis of significance of stalk rot score on average (SRS_A_) (**C**) and disease severity index (DSI) (**D**) between ZC17 and CH72. The symbol “***” indicates significant differences (*p* < 0.001, *n* = 5) between two groups, based on Student’s *t*-test.

As described previously, we inoculated maize inbred lines CH72 and ZC17 at leaf nine. At 7 days post inoculation, the inoculated internodes were divided to observe in detail the *F. proliferatum* infestation and the appearance of stalk symptoms among the different groups. Obviously, compared with the mock-inoculation group, the *F. proliferatum* inoculation group showed obvious symptoms in the stalks, with brown areas in the upper and lower nodes of the inoculation site and darker areas closer to the wound location. More than half of the inoculated nodes showed typical dark brown symptoms, and even the lesion tissues in the vicinity of the wounds had completely collapsed and decayed (Figure 1). The inoculated group of samples from the resistant inbred line ZC17 showed significantly lower lesion areas and symptoms than those of the sensitive inbred line CH72 (Figure 1A,B). The SRS_A_ and DSI of the ZC17 and CH72 stalks were consistent with the symptom phenotypes: the susceptible inbred CH72 had approximately 2.48-fold more SRS_As_ than the resistant inbred ZC17 line (Figure 1C), and its DSI increased by 35.36% compared to that of ZC17 (Figure 1D).

### 2.2. Transcriptome Analysis and DEG Screening of CH72 and ZC17 Stems Inoculated with F. proliferatum

**Figure 2:**
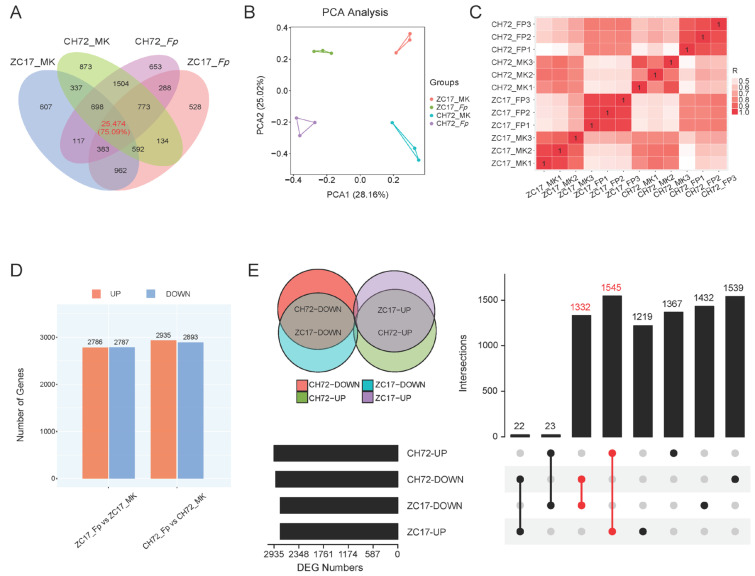
Analysis of transcriptome sequencing data. (**A**) Venn diagram of the number of expressed genes in the four groups of samples. (**B**) Principal component analysis (PCA) based on gene expression profiles for the four groups of samples. (**C**) Heatmap of the correlation of gene expression for all samples. (**D**) Histograms of the number of up- and down-regulated DEGs in the *F. proliferatum*-inoculated group compared to the mock-inoculated group in the two inbred lines. (**E**) The Upset plot demonstrates the DEGs that were jointly up-regulated and jointly down-regulated in the two inbred lines (ZC17 and CH72) after *F. proliferatum* inoculation.

To investigate the gene expression patterns of CH72 and ZC17 after *F. proliferatum* inoculation, we sequenced the transcriptomes of two maize inbred lines with three biological replicates from each group for mock inoculation and *F. proliferatum* inoculation. The cDNA libraries were constructed and sequenced using the Illumina NovaSeq 6000 platform. A total of 529,479,124 raw reads were generated from the 12 samples, and 515,206,860 high-quality clean reads were identified after filtering. The average number of clean reads per sample was 429,339,905, and the average valid ratio was 97.30%. The average alignment rate with the reference maize genome B73_V4 was 89.73%, and the average percentages of the sequencing quality indexes Q20 and Q30 were 99.95% and 98.72%, respectively, indicating the stable performance of the sequencer and the high quality of the sequencing data (Table S1). In the *Fp* and MK groups of CH72, 29,890 and 30,385 genes were identified from three replicates, respectively, and 29,134 and 29,071 genes were identified in the *Fp* and MK groups of ZC17, respectively. In addition, 25,474 genes were coidentified across the four groups, accounting for 75.09% of the total (Figure 2A). The results of the principal component analysis (PCA) showed a high reproducibility of the samples within the group. The different groups were separated from each other (Figure 2B). In PC1 (28.16%), the *Fp* and MK groups were segregated from each other. In PC2 (25.02%), ZC17 and CH72 were separated from each other. The correlation heatmap between all samples was consistent with the PCA results (Figure 2C). These analyses demonstrated that the RNA sequencing (RNA-Seq) data exhibited high levels of reliability and reproducibility.

Furthermore, to investigate the gene expression changes induced by *F. proliferatum* infection in CH72 and ZC17, we screened two inbred lines for differentially expressed genes (DEGs) between the *F. proliferatum*-inoculated and mock-inoculated groups. The DEGs in the two comparison pairs (ZC17_Fp vs. ZC17_MK, CH72_Fp vs. CH72_MK) were analyzed (Figure 2D). In ZC17, there were 5573 DEGs (2786 up-regulated and 2787 down-regulated) compared to its MK. Correspondingly, in CH72, there were 5828 DEGs (2935 up-regulated and 2893 down-regulated). After that, we took the intersecting up- and down-regulated DEGs obtained in the two comparison groups and were surprised to find that the number of jointly up-regulated and jointly down-regulated DEGs in the comparison groups of the two inbred lines were 1545 and 1332, respectively. The number of DEGs that were up-regulated in ZC17 and down-regulated in CH72 and the number that were down-regulated in ZC17 and up-regulated in CH72 were only 22 and 23, respectively (Figure 2E). Therefore, we hypothesized that the mechanism of response to *F. proliferatum* infestation in these two maize inbred lines with different resistances might be similar, and we focused our attention on the genes that were jointly up- and down-regulated in the two inbred lines.

### 2.3. Functional Enrichment of DEGs in Response to F. proliferatum Infestation in Maize

**Figure 3:**
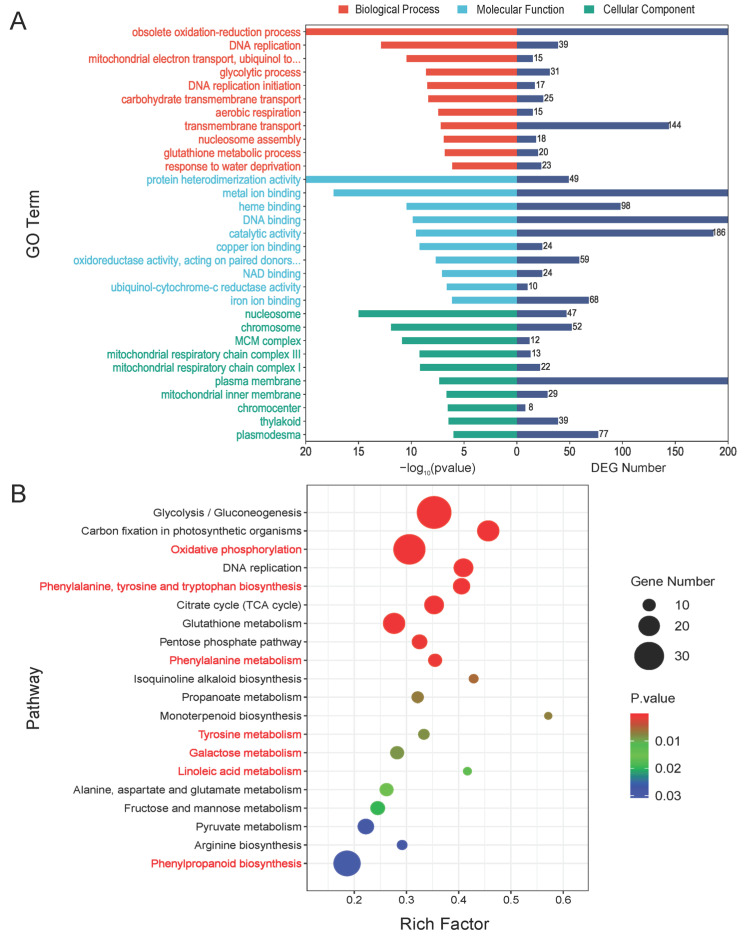
The GO and KEGG enrichment analyses of DEGs. (**A**) A histogram of the GO enrichment analysis with the top 10 entries with the smallest *p*-value selected for each Biological Process (BP), Cellular Component (CC), and Molecular Function (MF). The left bar is −log(*p*-value) and the right bar is the number of DEGs for the corresponding entry. (**B**) A bubble diagram of the KEGG enrichment analysis of differential genes. Pathways colored in red are closely related to plant disease resistance.

To investigate the function of the shared DEGs between the two comparison groups, we annotated the DEGs using the Gene Ontology (GO) database and performed an enrichment analysis. In Figure 3A, we show the top 10 GO terms with the smallest *p* values that were significantly enriched in the categories of Biological Process (BP), Cellular Component (CC), and Molecular Function (MF). The GO term with the smallest *p* value in the BP classification was the obsolete oxidation-reduction process (GO:0055114), with a DEG count of 300. The next term was DNA replication (GO:0006260), with 39 DEGs. There were also 144 DEGs enriched in transmembrane transport (GO:0055085). The terms with the highest number of DEGs in MF were DNA binding (GO:0003677), metal ion binding (GO:0046872), and catalytic activity (GO:0003824), with 281, 266, and 186 DEGs, respectively. In contrast, the term with the highest number of differential genes in CC was the plasma membrane (GO:0005886), with 211 DEGs, suggesting that *F. proliferatum* has a relatively large impact on genes localized to the plasma membrane (Table S2). Subsequently, we compared the annotations of up- and down-regulated DEGs in the GO database, and the most down-regulated DEGs in the GO terms of BP were involved in the regulation of DNA-templated transcription (GO:0006355), transmembrane transport (GO:0055085), and DNA-templated transcription (GO:0006351), while up-regulated DEGs were more involved in obsolete oxidation-reduction processes (GO:0055114) and metabolic processes (GO:0008152), implying that up- and down-regulated DEGs may initiate different functional mechanisms when infested by *F. proliferatum*. The results were similar in MF and CC (Figure S1).

The Kyoto Encyclopedia of Genes and Genomes (KEGG) pathway analysis indicated that most DEGs were annotated to ribosomes (ko03010), glycolysis/gluconeogenesis (ko00010), oxidative phosphorylation (ko00190), phenylpropanoid biosynthesis (ko00940), plant hormone signal transduction (ko04075), carbon fixation in photosynthetic organisms (ko00710), and glutathione metabolism (ko00480) (Table S3). The KEGG enrichment analysis results identified the top 20 pathways with the lowest *p*-values (Figure 3B). The results showed that oxidative phosphorylation (zma00190), phenylalanine, tyrosine, and tryptophan biosynthesis (zma00400), phenylalanine metabolism (zma00360), tyrosine metabolism (zma00350), galactose metabolism (zma00052), linoleic acid metabolism (zma00591), and phenylpropanoid biosynthesis (zma00940), which have been previously reported to be associated with plant disease resistance, were significantly enriched. Then, clustered heatmaps of the key DEGs in each of these pathways were drawn and it was shown that most of them were activated after inoculation (Figure S2). In addition, we found that up-regulated DEGs and down-regulated DEGs differed significantly in the KEGG pathway (Figure S3). The KEGG level 1 classification had the most up-regulated DEGs belonging to the metabolism, with the top five pathways being phenylpropanoid biosynthesis (ko00940), glycolysis/gluconeogenesis (ko00010), carbon fixation in photosynthetic organisms (ko00710), glutathione metabolism (ko00480), and oxidative phosphorylation (ko00190). The pathways with the highest number of down-regulated DEGs were plant hormone signal transduction (ko04075), MAPK signaling pathway-plant (ko04016), ubiquitin-mediated proteolysis (ko04120) and circadian rhythm-plant (ko04712), corresponding to KEGG level 1 for environmental information processing, genetic information processing, and organismal systems, respectively. These results suggested that up- and down-regulated DEGs were involved in different pathways to defend against *F. proliferatum* invasion.

### 2.4. Transcription Factor (TF) Family Analysis

**Figure 4:**
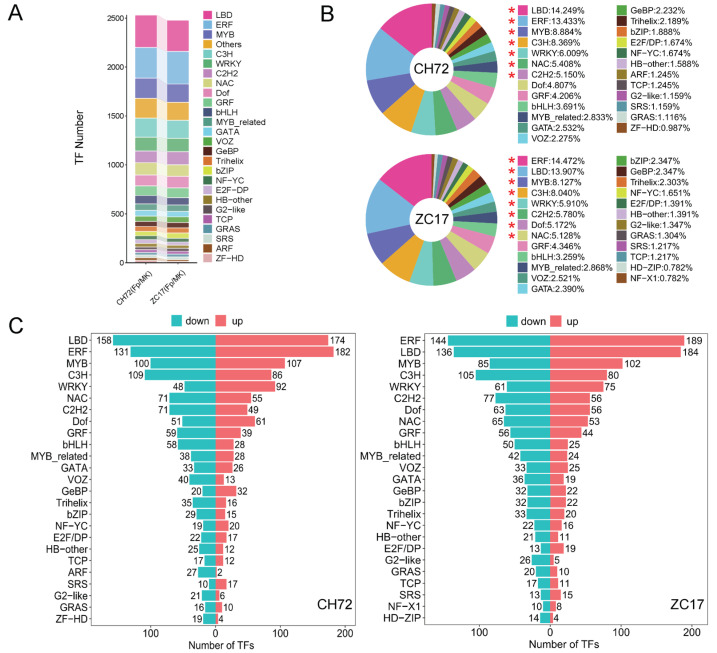
Analysis of transcription factors. (**A**) A bar-stacked plot of the top 25 TF families with the highest numbers in ZC17 and CH72. (**B**) The top 25 TF families with the highest numbers in ZC17 and CH72 and their percentages are illustrated in this circular graph. Those marked with “*” indicate TF families with more than 5% in each of the two inbred lines. (**C**) The bar graph shows the number of up- and down-regulations in the top 25 TF families. Blue indicates down-regulation and red indicates up-regulation.

As we know, a large number of studies have reported that transcription factors play important roles in plant disease resistance as well as in responses to environmental stresses. There were 2537 and 2483 DEGs in CH72 (*Fp* vs. MK) and ZC17 (*Fp* vs. MK), respectively, which were further analyzed and identified as TFs, and these DEGs belonged to 50 TF families, mainly including LBD, ERF, MYB, C3H, WRKY, NAC, C2H2, Dof, GRF, and bHLH (Figure 4A, Table S4). To further explore the alteration of the TF families in CH72 and ZC17 under the impact of *F. proliferatum*, we showed the top 25 TF families with the highest percentage of expression in the two inbred lines (Figure 4B), and the results showed that the affected TF families and their proportions were basically the same. In addition, we found that the number of up-regulated DEGs was higher than that of down-regulated DEGs for TF families such as LBD, ERF, MYB, and WRKY, while the number of down-regulated DEGs was higher than that of up-regulated DEGs for most of the other TF families, such as C3H, C2H2, NAC, bHLH, and bZIP, which have been previously reported to be significantly associated with plant disease resistance (Figure 4C). The results of the transcription factor analysis were similar to those of the DEG enrichment analysis, further confirming our speculation that CH72 and ZC17 may respond through the same pathways when infested with *F. proliferatum*. Clustering heatmaps of the bHLH, MYB, NAC, and WRKY families were drawn, demonstrating the important role of transcription factors in the maize response to *F. proliferatum* infestation (Figure S4).

### 2.5. Metabolite Identification and DEM Analysis of CH72 and ZC17 after Infestation with F. proliferatum

**Figure 5:**
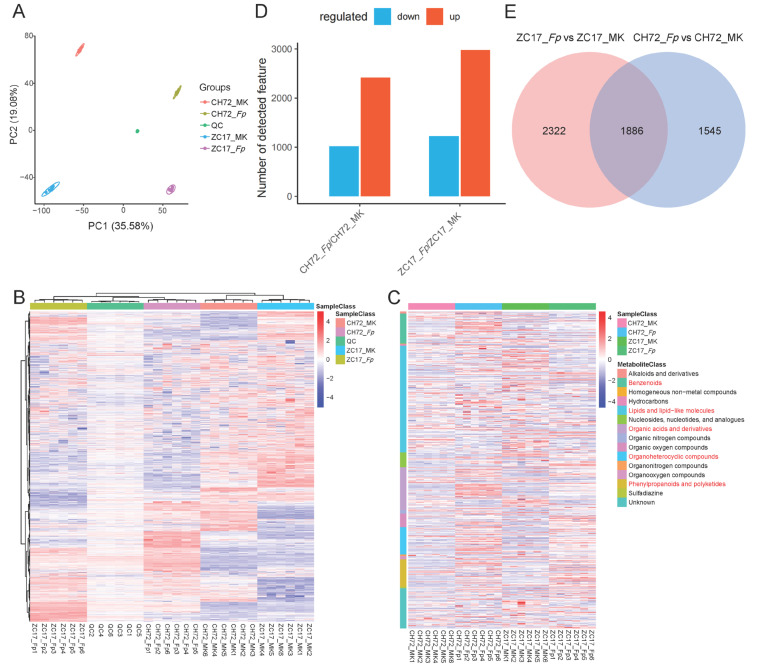
Overview analysis of metabolomics. (**A**) PCA analysis of different groups of metabolites. (**B**) Heatmap of clustering of identified metabolic features in all samples. The rows are clustered for all metabolic features and the columns are clustered for all samples. (**C**) Heatmap of the annotated classifications of all substances identified in the secondary mass spectra. Substance classifications with a high percentage are marked in red. (**D**) Histogram of difference analysis of metabolic features identified in ZC17 and CH72. (**E**) Venn diagrams of differential metabolic features in two inbred lines.

In the metabolomic data, we identified 9143 and 8257 ion (ESI+ and ESI−) modes, respectively. In addition, a total of 409 and 250 metabolites were identified in the anionic and cationic modes of LC-MS secondary mass spectrometry, respectively. The PCA results showed that the metabolome was strongly similar to the transcriptome data, with *Fp* and MK separated from each other in PC1 (35.58%), indicating a significant difference in the metabolite levels between the *Fp* and MK groups, and CH72 and ZC17 separated from each other in PC2 (19.08%), indicating that the metabolite levels between the two inbred lines also differed but were smaller than those brought about by *Fp* inoculation (Figure 5A). Furthermore, PCA score plots showed that the near pooling of the mixed group samples used for quality control (QC) demonstrated remarkable experimental repeatability and that the samples within the same group were close together and groups were separated from each other, indicating that the metabolome data in this study were accurate and reliable. Subsequently, we plotted a hierarchical clustering heatmap for all the identified metabolite features, which showed a high correlation of samples within the group, and that the clustering results were consistent with the PCA results (Figure 5B). The heatmap results of metabolites identified by secondary mass spectrometry showed that the main metabolite classifications were benzenoids, lipids and lipid-like molecules, organic acids and their derivatives, organoheterocyclic compounds, and phenylpropanoids and polyketides, which have been previously reported to be closely related to plant disease resistance (Figure 5C).

Next, we analyzed the identified metabolic features for significant differences and, in this study, we used a univariate analysis of the multiplicity of differences (fold-change) and *t*-test, combined with the variable important for the projection (VIP) value obtained by the multivariate statistical analysis PLS-DA, when screening for DEMs. The differentially expressed features satisfy the following three conditions at the same time: (1) a ratio ≥ 2 or ratio ≤ 1/2; (2) *p* value < 0.05; and (3) VIP ≥ 1. In CH72 (*Fp* vs. MK), 2415 and 1016 metabolic features were significantly up- and down-regulated, respectively. Correspondingly, in ZC17 (*Fp* vs. MK), the numbers of significantly up- and down-regulated metabolite features were 2980 and 1228, respectively (Figure 5D). A total of 1886 significantly different metabolic features were common to both comparison groups. The number of significantly different metabolite features that appeared in ZC17 (*Fp* vs. MK) or CH72 (*Fp* vs. MK) alone was 2322 and 1545, respectively (Figure 5E).

**Figure 6:**
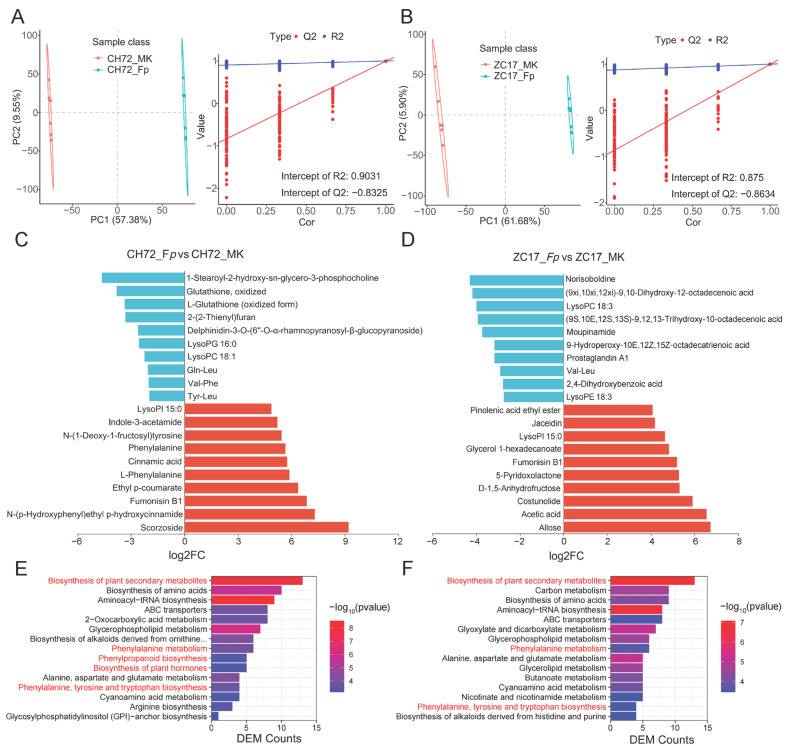
Differential metabolite analysis and functional enrichment. The PLS-DA analysis and the validation analysis of the permutation test in CH72 (**A**) and ZC17 (**B**). Histograms of the top 10 metabolites with the largest fold change in terms of up- and down-regulated differences in CH72 (**C**) and ZC17 (**D**). The KEGG pathway enrichment analysis bar graphs showed the top 10 pathways that were most significantly enriched (with the smallest *p*-value) in CH72 (**E**) and ZC17 (**F**), respectively, in reverse order of the number of DEMs.

To validate the accuracy of the differentially abundant metabolite analysis, we then conducted a partial least square discriminant analysis (PLS-DA). The analysis of the results of the PLS-DA model for CH72 and ZC17 showed a significant difference in the first component (horizontal axis) at cross-validation, implying a separation between groups. At the same time, the PLS-DA model parameters R2 and Q2 were subjected to a permutation test, and the number of tests was set to 200. The R2 and Q2 values of the CH72 (*Fp* vs. MK) permutation test plot were 0.9031 and −0.8325, respectively, which indicated that the current PLS-DA model was more reliable (Figure 6A). Similarly, the R2 and Q2 values of the ZC17 (*Fp* vs. MK) permutation test plot were 0.875 and −0.8634, respectively (Figure 6B). Q2 < 0 indicated that the model was not overfitted and that the results of the differentially abundant metabolite analysis were accurate.

This study also identified the 10 metabolites with the greatest up- and down-regulated differences between each comparison group based on the level of change in the differentially abundant metabolites (Figure 6C,D). Phenylalanine, cinnamic acid, ethyl p-coumarate, indole-3-acetamide, and N-(1-deoxy-1-fructosyl) tyrosine were significantly up-regulated in CH72. In contrast, jaceidin (a flavonoid), glycerol 1-hexadecanoate, polysaccharides, and lipids were significantly elevated in ZC17. In addition, LysoPI 15:0 was significantly elevated in both ZC17 and CH72. The largest number of these DEMs belong to lipids and lipid-like molecules (15), followed by organic acids and their derivatives (12), and a few belong to organoheterocyclic compounds (4), phenylpropanoids and polyketides (4), and benzenoids (2). The key metabolic pathways of maize that respond to *F. proliferatum* were further identified by the KEGG enrichment pathway analysis of DEMs. As shown in Figure 6E,F, 15 pathways were selected from the KEGG database that were most highly enriched (with the smallest *p* value) in CH72 and ZC17. In CH72 (*Fp* vs. MK), some pathways associated with plant disease resistance were significantly enriched, such as the biosynthesis of plant secondary metabolites; phenylalanine metabolism; phenylpropanoid biosynthesis; biosynthesis of plant hormones; and phenylalanine, tyrosine, and tryptophan biosynthesis (Figure 6E). In addition, the biosynthesis of plant secondary metabolites; phenylalanine metabolism; and phenylalanine, tyrosine, and tryptophan biosynthesis were also significantly enriched in ZC17 (*Fp* vs. MK) (Figure 6F). Thus, metabolites in these pathways may play an important role in the maize response to *F. proliferatum*.

### 2.6. Integrated Analysis of Transcriptomics and Metabolomics Data Reveals the Mechanisms of the Maize Response to F. proliferatum

**Figure 7:**
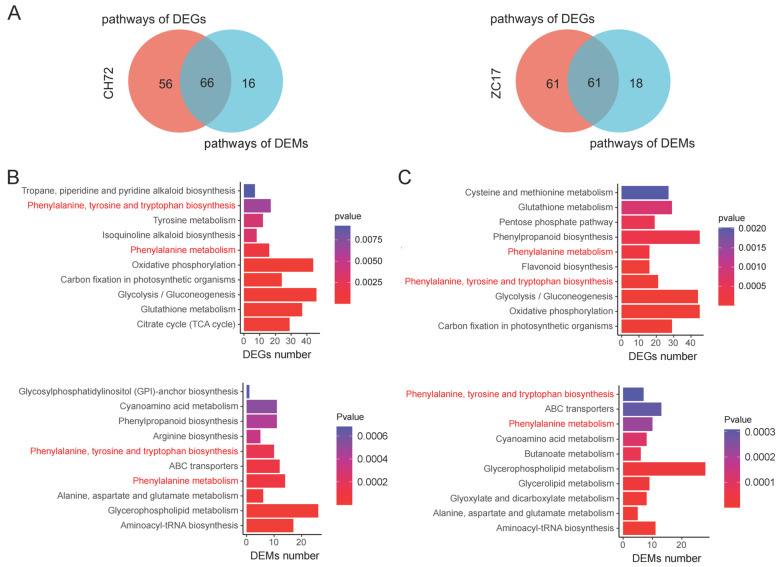
Integrated analysis of the transcriptome and metabolome. (**A**) Pathways involved in differential genes and differential metabolites in CH72 and ZC17 were intersected by Venn diagrams. The top 10 pathways that were most significantly enriched in each of the differential genes and differential metabolites among the pathways that intersect CH72 (**B**) and ZC17 (**C**) are shown as bar graphs. There are two pathways labeled in red that are co-enriched in the four pathway enrichment bar graphs.

We then integrated transcriptomic and metabolomic data to further understand the mechanisms by which maize responds to *F. proliferatum*. The PCA results of the transcriptome and metabolome were highly consistent, with *Fp* separated from MK in the first principal component and CH72 separated from ZC17 in the second principal component. Then, DEGs and DEMs were simultaneously localized to the KEGG pathway map to better assess the relationship between the genes and metabolites. In CH72 (*Fp* vs. MK), 66 pathways were shared by DEGs and DEMs, whereas 61 pathways were common in ZC17 (*Fp* vs. MK) (Figure 7A). In Figure 7B,C, we show the 10 pathways most significantly enriched in terms of DEGs and DEMs of the pathways shared by CH72 and ZC17 with the smallest *p* values. Interestingly, only phenylalanine, tyrosine, and tryptophan biosynthesis and phenylalanine metabolism were significantly enriched in both the transcriptomic and metabolomic data for CH72 and ZC17. This result suggested that these two pathways play a key role in the maize response to *F. proliferatum*.

**Figure 8:**
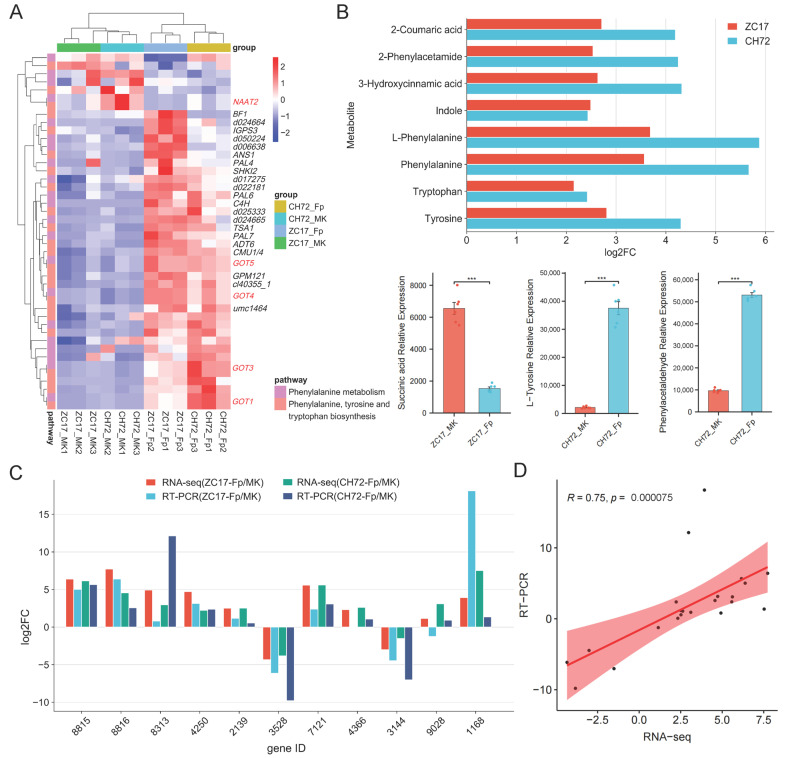
Further analysis of the phenylalanine, tyrosine, and tryptophan biosynthesis and phenylalanine metabolism pathways and their qPCR validation. (**A**) Heatmap of the clustering of DEGs involved in the phenylalanine, tyrosine, and tryptophan biosynthesis and phenylalanine metabolism pathways. DEGs involved in both pathways are marked in red. (**B**) Bar graphs and histograms of the expression trends of DEMs in these two pathways in ZC17 and CH72. The symbol “***” indicates significant differences (*p* < 0.001, *n* = 6) between two groups, based on Student’s *t*-test. (**C**) Histogram of the fold change of DEGs in transcriptome sequencing and qPCR results. (**D**) Spearman rank correlation analysis showed high concordance between the RNA-seq and qPCR results.

**Figure 9:**
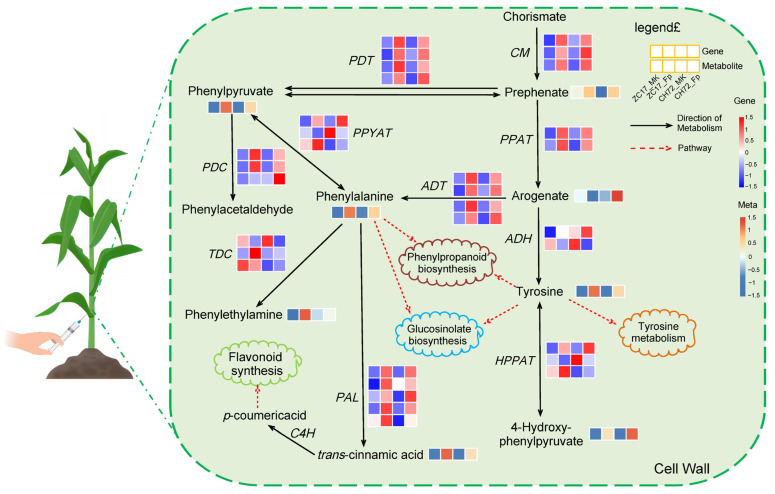
The schematic diagram of key genes and metabolites in the phenylalanine metabolic pathway involved in maize the response to *F. proliferatum*. CM, chorismate mutase; PDT, prephenate dehydratase; PPAT, prephenate aminotransferase; ADH, arogenate dehydrogenase; HPPAT, 4-hydroxyphenylpyruvate aminotransferase; ADT, arogenate dehydratase; PPYAT, phenylpyruvate aminotransferase; PDC, p-coumaric acid decarboxylase; TDC, tyrosine decarboxylase; PAL, phenylalanine ammonia lyase; C4H, cinnamate 4-hydroxylase.

As shown by the results of transcriptome sequencing, most of the DEGs in these two pathways were significantly up-regulated, and we hypothesized that these two pathways were activated in maize stems under the influence of *F. proliferatum* to defend against invasion. Further analysis of the DEGs in these two pathways showed that five key genes were involved in both pathways, *Zm00001d048736* (*NAAT2*), *Zm00001d010190* (*GOT5*), *Zm00001d042685* (*GOT4*), *Zm00001d016198* (*GOT3*), and *Zm00001d043382* (*GOT1*) (Figure 8A). The DEMs identified in the phenylalanine metabolism and phenylalanine, tyrosine, and tryptophan biosynthesis pathways were similar to the transcriptome results, with the majority being significantly up-regulated. Eight of these metabolites showed consistent expression trends in CH72 and ZC17, namely, 2-coumaric acid, 2-phenylacetamide, 3-hydroxycinnamic acid, indole, L-phenylalanine, phenylalanine, tryptophan, and tyrosine. Succinic acid was significantly reduced in abundance in the *Fp* group compared to the MK group in ZC17. In addition, the abundance of L-tyrosine and phenylacetaldehyde was significantly higher only in CH72 (Figure 8B). To further confirm the reliability of the sequencing data, we randomly selected 11 genes for qPCR validation, which showed that the trends of the RNA-seq and qPCR results were consistent in both CH72 and ZC17 (Figure 8C). Spearman rank correlation analysis also showed high concordance (R = 0.75, *p* = 7.5 × 10^−5^) between the RNA-seq and qPCR results, indicating the high accuracy of the sequencing data (Figure 8D). Combining DEGs and DEMs, the role of the phenylalanine metabolism pathway (zma00360) and the phenylalanine, tyrosine, and tryptophan biosynthesis pathway (zma00400) in maize stalk rot resistance was schematically mapped based on the KEGG database (Figure 9). It is evident from the diagrams that genes and metabolites related to phenylalanine were activated in response to *F. proliferatum*. In addition, this also demonstrated the important role of these genes and metabolites, in maize, against stalk rot.

## 3. Discussion

It has been reported that various types of plant diseases caused by pathogens cause significant losses in the yield and quality of important crops around the world every year [30]. *Fusarium* spp. can cause significant damage to a wide range of tissues in maize, and, in the past, it has been studied more in terms of ear and grain rot. In recent years, with the rapid development of mechanized harvesting, research on stem rot has received increasing attention. The selection of resistant varieties is the most economical and environmentally friendly way to prevent and control stalk rot. However, progress in the screening of resistance genes and the molecular mechanisms of host disease resistance is still limited, and it will become increasingly important to explore internal resistance genes to improve maize resistance. To this end, in this study, we conducted transcriptome and metabolome analyses of ZC17 (resistant) and CH72 (susceptible) maize plants infected with *F. proliferatum*. Our results showed that ZC17 displayed a significantly higher resistance to *F. proliferatum* infection than CH72 (Figure 1). Transcriptome analysis showed that the number of DEGs was higher in the susceptible inbred line CH72 than in the resistant inbred line ZC17, implying that the infection pressure on CH72 was higher than that on ZC17. This is similar to the results obtained in other previous studies on disease resistance, and the reason for the results may be that the tissues of the sensitive material are more easily invaded by the pathogen, which causes more damage and thus requires the expression of more transcriptional regulatory genes to adapt to the infestation [31,32]. The number of DEGs that were jointly up-regulated in ZC17 and CH72 was 1545, and the number of DEGs that were jointly down-regulated was 1332. This result implies that maize-resistant and maize-susceptible inbred lines have similar response mechanisms to *F. proliferatum* infestation (Figure 2E). GO enrichment analysis showed that the genes coregulated in the two inbred lines were mainly involved in phosphorylation, metabolic processes, and the alteration of enzyme activities (Figure S1). KEGG results showed that the pathways associated with plant stress tolerance were significantly up-regulated, including oxidative phosphorylation; phenylalanine, tyrosine, and tryptophan biosynthesis; and linoleic acid metabolism, among others (Figure S3). In addition, the KEGG enrichment analysis of differentially abundant metabolites showed that, similar to the transcriptome results, differentially abundant metabolites were mainly enriched in the biosynthesis of plant secondary metabolites, phenylpropanoid synthesis, and the biosynthesis of plant hormones (Figure 6). Furthermore, combined transcriptomic and metabolomic analyses identified phenylalanine metabolism and phenylalanine, tyrosine, and tryptophan biosynthesis as two key pathways that were screened out, implying that phenylalanine plays an important role in maize resistance to *F. proliferatum* infestation (Figure 7).

### 3.1. Phenylalanine Metabolism Is Important for Plant Disease Resistance

Phenylalanines are very important secondary metabolites for plants and could be involved in a range of plant responses to pathogens [33]. In response to pathogen infection, key enzymes in the phenylalanine pathway such as phenylalanine ammonia-lyase (PAL), peroxidase (POD), and polyphenol oxidase (PPO) are involved in lignin synthesis, which provides a structural barrier for the plant against the pathogen. Furthermore, phenylalanine is deaminated to form cinnamic acid by the action of PAL (Figure 9). Cinnamic acid forms 4-coumaroyl-CoA due to the action of cinnamic acid-4-hydroxylase (C4H) and 4-coumarate-CoA ligase (4CL). The products are subsequently catalyzed by different enzymes, such as chalcone synthase (CHS) and chalcone isomerase (CHI), to form a series of flavonoids. All compounds with the basic structural backbone of flavonoids share this common biosynthetic pathway, and flavonoids play an important role in the plant response to pathogens [34]. Flavonoids induce callose and tylose synthesis and crystal structure formation, thereby minimizing pathogen expansion by shutting down vascular tissue [35]. In this study, we found that the 26 up-regulated DEGs during *F. proliferatum* infection were enriched in the phenylalanine metabolism (11) and phenylalanine, tyrosine, and tryptophan biosynthesis (15) pathways, including C4Hs, PALs, ADTs, GOTs, and other key enzymes (Figure 8A). In addition, the metabolites that were significantly up-regulated in these two pathways have very important roles in plant disease resistance, such as 2-coumaric acid, 3-hydroxycinnamic acid, indole, phenylalanine, tryptophan, and tyrosine (Figure 8B). Previous studies have reported the role of coumaric acid and hydroxycinnamic acid in inhibiting fungal growth and promoting resistance to *Fusarium* head blight infection [36]. Phenylalanine, tryptophan, and tyrosine are very crucial in plant metabolic pathways, synthesized from precursors of the shikimate pathway [37]. And the shikimate pathway plays an important role in various biological processes in plants, such as growth and development and resistance to stress. The biosynthetic pathways of these aromatic amino acids are precursors for the synthesis of phytohormones and aromatic secondary metabolites, which play a crucial role in plant disease resistance [38].

### 3.2. The Role of TFs in the Maize Response to F. proliferatum Infestation

Plant TFs play an important role in the PTI process and sector-triggered immunity [39]. Plants activate transcription factors in response to external stimuli through a series of signals that can regulate plant disease resistance either alone or by modulating the expression of downstream defense-related genes. MYB is reported to be one of the most important families of transcription factors in plants, and it can be involved in regulating a wide range of biological processes in plants [40]. Recent studies have reported that upon sensing jasmonic acid signaling in maize, *ZmJAZs* release *ZmMYC7*, which activates the expression of *ZmERF147* by binding to the G-box in the promoter region of *ZmERF147*, which further activates the expression of downstream *ZmPRs*, thereby enhancing resistance to pathogens [41]. It has also been shown that the transcription factors C2H2 and MYB were able to coordinate the reduction in H_2_O_2_ degradation in rice to improve its resistance to rice blast [42]. Another study showed that the overexpression of *TaPIMP1*, a transcription factor of R2R3-MYB, enhances the resistance to *Bipolaris sorokiniana* in wheat [43]. In the present study, after inoculation with FP, we found some significantly changed MYB-family transcription factors compared with the mock-inoculated group, and the expression patterns of these MYB-family transcription factors were similar in ZC17 and CH72 (Figure S4). The NAC transcription factor family also has a very important role in plant growth and development. Previous studies have shown that many NAC TFs were induced in plants in response to pathogens [44]. For example, *OsNAC19* and *OsNAC111* were induced to be expressed when rice was infected by *Magnaporthe oryzae*, and thus participated in the defense response against rice blast [45,46]. Mitogen-activated protein kinase (MPK) is a key regulator of plant antioxidant defense systems under various stimuli. Recent studies have shown that *ZmMPK5* can enhance the antioxidant capacity of maize by phosphorylating *ZmNAC49*, increasing the expression of *ZmSOD3* and enhancing SOD activity [47]. In the present study, we found that the number of DEGs belonging to the NAC family in CH72 was slightly higher, at 126, than the 118 in ZC17, and the ratio of the number of up- to down-regulated genes was nearly equal in the two inbred lines. We hypothesized that the up-regulated NAC TFs may be positive regulators of maize stalk rot, while the down-regulated TFs were negative regulators. How NAC family members regulate stalk rot in maize remains to be further investigated.

The WRKY family contains conserved WRKY structural domains, which are not only involved in physiological processes such as plant seed germination, pollen development, and plant senescence, but also play crucial roles in defense responses such as disease and stress resistance in plants [48]. Previous studies have confirmed that WRKY TFs have a crucial role in the maize response to environmental stress. For example, *ZmWRKY17* regulates maize sensitivity to ABA, thereby modulating its resistance to salt stress [49]. *ZmWRKY106* plays a positive role under drought and high-temperature stress [50]. On the other hand, WRKYs have been increasingly studied in maize-responsive pathogens. Recent studies suggest that *ZmWRKY52*, *ZmWRKY71*, and *ZmWRKY83* may have important contributions to the maize resistance to *Aspergillus flavus* [51]. In another study, *ZmWRKY83* positively regulated maize resistance to *F. graminearum* [52]. In addition, bHLH and MYB form a complex that participates in the secondary metabolism of flavonoids and anthocyanins, increasing plant resistance to pathogens [53]. These findings provide a foundation for the potential molecular mechanism of maize resistance to stalk rot.

In our next study, we need to place more emphasis on studying the interactions between maize and *F. proliferatum*. In brief, we need to study how *F. proliferatum* evades the immune response of maize and how maize recognizes *F. proliferatum* infection and initiates a series of defense responses, mining key genes and revealing the complex regulatory mechanisms of maize disease resistance. Of particular importance in this study is the comprehensive analysis of the phenylalanine metabolic pathway in maize stalk rot resistance, which was determined by a combination of phenotypic, transcriptomic, and metabolomic techniques. In addition, it was shown that transcription factors play an important role in maize disease resistance. This will provide a valuable theoretical basis for deepening our understanding of the mechanisms involved in the maize resistance to stalk rot caused by *F. proliferatum*.

## 4. Materials and Methods

### 4.1. Sources of Materials, Method of Inoculation, and Assessment of Symptoms

The *F. proliferatum* strain HB24-3-1 used in this study was obtained from Henan Academy of Agricultural Sciences. This strain was cultured on fresh potato dextrose agar (PDA) plates and the conditions were set as 25 °C for 5 to 7 days in the dark. The inoculum was prepared by thoroughly mixing five flourish hyphal mats with a volume of about 125 mL using a kitchen blender and then adjusting to a final volume of 200 mL with sterilized ddH_2_O. Maize inbred lines, ZC17 (ZhengC17, resistant) and CH72 (Chang72, sensitive), with different levels of resistance to *F. proliferatum* were grown in the same greenhouse in Zhengzhou (Henan Academy of Agricultural Sciences). The resistant inbred line ZC17 has a plant height of 175–180 cm. The whole plant has 20–21 leaves, upswept leaves, dark green leaf color, and strong disease resistance. Sensitive inbred line CH72 has a plant height of 170–180 cm, spike height of 80 cm, main stem leaf number of 19, and is a compact plant, leaves upswept, male spike developed, weak disease resistance. The condition parameters of temperature and light in the greenhouse and the cultivation conditions were consistent with those described in our previous studies [19]. The maize inbred line germination experiment was conducted in September 2022, and the inoculation experiment was performed in October. The maize plant inoculation method was based on previous studies with minor modifications. Briefly, at the 9th leaf stage, plants were selected based on their similarity in the performance for inoculation to avoid environmental influence. Holes were punched at the second or third internodes of the maize plants with a sterile syringe tip and then the plants injected with 50 μL of *F. proliferatum* inoculum. The mock-inoculated group was injected with an equal amount of PDA as a control. All plants were closed with Vaseline after inoculation. At 7 days post inoculation, the upper and lower stem segments of the inoculated area were sampled. Nine plants were taken from each group and quick-frozen in liquid nitrogen for 1 h, after which they were transferred to a −80 °C refrigerator for storage. Three of the biological replicates had their RNA extracted for transcriptome sequencing and the other six biological replicates for metabolomics.

At 7 days post inoculation, internodes of isolated inoculated *F. proliferatum* and mock-inoculated maize plants were evaluated for maize stalk rot symptoms according to methods described in previous studies, and the stalk rot score on average (SRS_A_) and disease severity index (DSI) were calculated.

### 4.2. The Method of RNA Extraction, Library Construction, and Sequencing

Total RNA from the samples was isolated and purified using TRIzol (Invitrogen, Carlsbad, CA, USA) according to the standard protocol provided by the manufacturer. The NanoDrop ND-1000 (NanoDrop, Wilmington, DE, USA) was used for quality control of the amount and purity of the total RNA. The RNA integrity was examined using a Bioanalyzer 2100 (Agilent, Santa Clara, CA, USA) and validated via a protocol of agarose electrophoresis. The RNA with a concentration > 50 ng/μL, RIN > 7.0, OD260/280 > 1.8, and a total amount > 1 μg was used for subsequent construction of the library. The qualified total RNA was fragmented after capturing mRNA with PolyA (polyadenylate), then reverse transcribed to synthesize cDNA, and then PCR was performed to form a 300 ± 50 bp library. Finally, we performed 2 × 150 bp paired-end sequencing (PE150) on an Illumina NovaSeq™ 6000 (LC-Bio Technology Co., Ltd., Hangzhou, China) following the vendor’s recommended protocol. The raw data have been deposited in the NCBI Sequence Read Archive (SRA) database, with the accession number PRJNA956666.

### 4.3. Bioinformatics Analysis of RNA-Seq Data

The raw data format of the sequencing was fastq, and the quality control of the raw data, including the removal of junctions, duplicate sequences, and low-quality sequences, was performed using fastp software (version 0.23.2) with default parameters. Then, the sequencing data were mapped to the reference genome (*Zea mays*, B73 RefGen_v4) using HISAT2 (ver.2.2.2.0) to obtain a bam format file. Genes or transcripts were assembled using StringTie software (version 1.3.4), and then transcript and gene expression levels were determined by calculating FPKM (fragments per kilobase of exon per million reads mapped). The DEGs were selected if they had a fold change > 2 or fold change < 0.5 via a parametric F test comparing nested linear models (*p* value < 0.05) using the R package edgeR (version 3.22.5). We mapped all the genes to terms in the Gene Ontology database and calculated the numbers of differentially enriched genes in each term. Using the cluster Profiler (v3.14.3) to perform GO enrichment analysis on the differential genes, the *p* value was calculated via the hypergeometric distribution method (the standard of significant enrichment was padj < 0.05), and the GO term with significantly enriched differential genes was obtained to determine the main biological functions performed by the DEGs. Similarly, we performed enrichment analyses of the KEGG pathways involved in differential genes, focusing on the significantly enriched pathways with a padj < 0.05.

### 4.4. Sample Extraction and Metabolome Analysis

The collected plant tissue samples were thawed on ice, and metabolites were extracted with 50% methanol buffer, and these supernatants were transferred into new 96-well plates to wait for mass spectrometry. In addition, pooled QC samples were also prepared by combining 10 μL of each extraction mixture. In addition, an equivalent amount of 10 μL of dilution was aspirated from each sample’s extraction mixture and mixed homogeneously as QC samples. All samples were acquired using the LC-MS system following machine orders. And all chromatographic separations were performed using an ultra-performance liquid chromatography (UPLC) system (SCIEX, UK). The reversed-phase separation was performed on an ACQUITY UPLC T3 column (100 mm × 2.1 mm, 1.8 µm, Waters, UK). The injection volume for each sample was 4 µL.

The metabolites eluted from the columns were detected using a high-resolution tandem mass spectrometer TripleTOF5600plus (SCIEX, Warrington, UK). The Q-TOF was operated in positive and negative ion modes, respectively. The ion spray voltage was set to 5000 V and −4500 V in the positive and negative ion modes, respectively. The mass spectrometry data were acquired in IDA mode. The TOF mass range was from 60 to 1200 Da. The mass accuracy was calibrated every 20 samples during the acquisition process. In addition, to assess the stability of the LC-MS throughout the acquisition process, a quality control sample (all pools) was collected after every 10 samples. Acquired MS data were preprocessed using XCMS software (version 3.2). LC-MS raw data files were converted into mzXML format and then processed by the XCMS, CAMERA and metaX toolbox implemented using R software (version 3.5.2). Each ion was identified by combining retention time (RT) and *m*/*z* data. The KEGG database was utilized to annotate the metabolites and an in-house fragment spectrum library of metabolites was used to further confirm the accuracy of the metabolite identifications. The intensity of peak data was further preprocessed by metaX and the preprocessed dataset was subjected to PCA analysis to detect outliers and assess batch effects. Student’s *t*-test was used to analyze the differences in metabolites between the two groups and the *p*-value was corrected using FDR (Benjamini-Hochberg). In addition, supervised PLS-DA was performed using metaX to distinguish different variables between groups to calculate VIP values. The VIP cutoff value was set to 1.0 for selecting important metabolic features.

### 4.5. Quantitative Real-Time PCR Analysis

For qRT-PCR, random hexamers were annealed to DNase-treated cDNA for reverse transcription using the PrimeScript^™^ RT reagent Kit with a gDNA Eraser (TaKaRa, Dalian, China) at 37 °C for 30 s. Then, cDNA was diluted 1:3 with ddH_2_O and stored at −20 °C. One microliter of diluted cDNA was used for qPCR in a Bio-Rad CFX96 Real Time System machine (Bio-Rad Laboratories Inc., Hercules, CA, USA). qRT-PCR was performed with 5Μ TB Green^®^ *Premix Ex Taq*^™^ II (Tli RNaseH Plus) (TaKaRa) in a 10 μL total reaction volume. Forward and reverse gene-specific primers used were designed by Primer Premier 5 software (Premier, Ottawa, ON, Canada) and are listed in Supplementary Table S5. GAPDH was used as an internal control. Three technical replicates were used for each sample. The relative mRNA abundance of each tested gene was averaged for triplicate reactions, and the values were normalized according to the Ct of the internal control using the 2^−ΔΔCt^ method [54].

### 4.6. Statistical Analysis

All data collected from phenotypic, physiological parameter, and RT–qPCR analyses were subjected to one-way variance analysis (ANOVA) and Student’s *t*-test using SPSS 22.0 software (IBM, New York, NY, USA). A *p* value < 0.05 indicates a significant difference, and a *p* value < 0.01 indicates a very significant difference. Bioinformatic analysis was also performed using the OmicStudio tools at https://www.omicstudio.cn/tool (accessed on 16 May 2023) [55].

## Data Availability

The RNA sequencing data have been deposited in the NCBI SRA (http://www.ncbi.nlm.nih.gov/sra, accessed on 22 March 2023) database, with the accession number PRJNA956666. The other referenced data are included in the article or the Supplementary Materials.

## Supplementary Materials

The following supporting information can be downloaded at https://www.mdpi.com/article/10.3390/ijms25031492/s1.
