# Supplementary material for: Transcriptomic and Metabolomic Analyses Reveal the Role of Phenylalanine Metabolism in the Maize Response to Stalk Rot Caused by Fusarium proliferatum
Source: Int J Mol Sci. 2024 Jan 25;25(3):1492. doi: 10.3390/ijms25031492 (PMC10855574; doi:10.3390/ijms25031492)
Supplement: Supplementary file 1 [file ijms-25-01492-s001.zip › Supplementary Figures.pdf]

## SUPPLEMEANTAL FILES

# Transcriptomic and Metabolomic Analyses Reveal the Role of Phenylalanine Metabolism in the Maize Response to Stalk Rot Caused by *Fusarium proliferatum*

**Jianjun Sun [1,†], Yanzhao Wang [1,†], Xingrui Zhang [2], Zeqiang Cheng [1], Yinghui Song [1], Huimin Li [1], Na Wang [1], Shen Liu [1], Zijia Cao [1], Hongxia Li [1], Wanying Zheng [1], Canxing Duan [2,*] and Yanyong Cao [1,3,*]**

[1] Institute of Cereal Crops, Henan Academy of Agricultural Sciences, Zhengzhou 450002, China
[2] Key Laboratory of Grain Crop Genetic Resources Evaluation and Utilization, Institute of Crop Sciences, Chinese Academy of Agricultural Sciences, Beijing 100081, China
[3] The Shennong Laboratory, Zhengzhou 450002, China
* Correspondence: duancanxing@caas.cn (C.D.); yanyongcao@126.com (Y.C.)
† These authors contributed equally to this work.

## SUPPLEMENTAL FIGURES

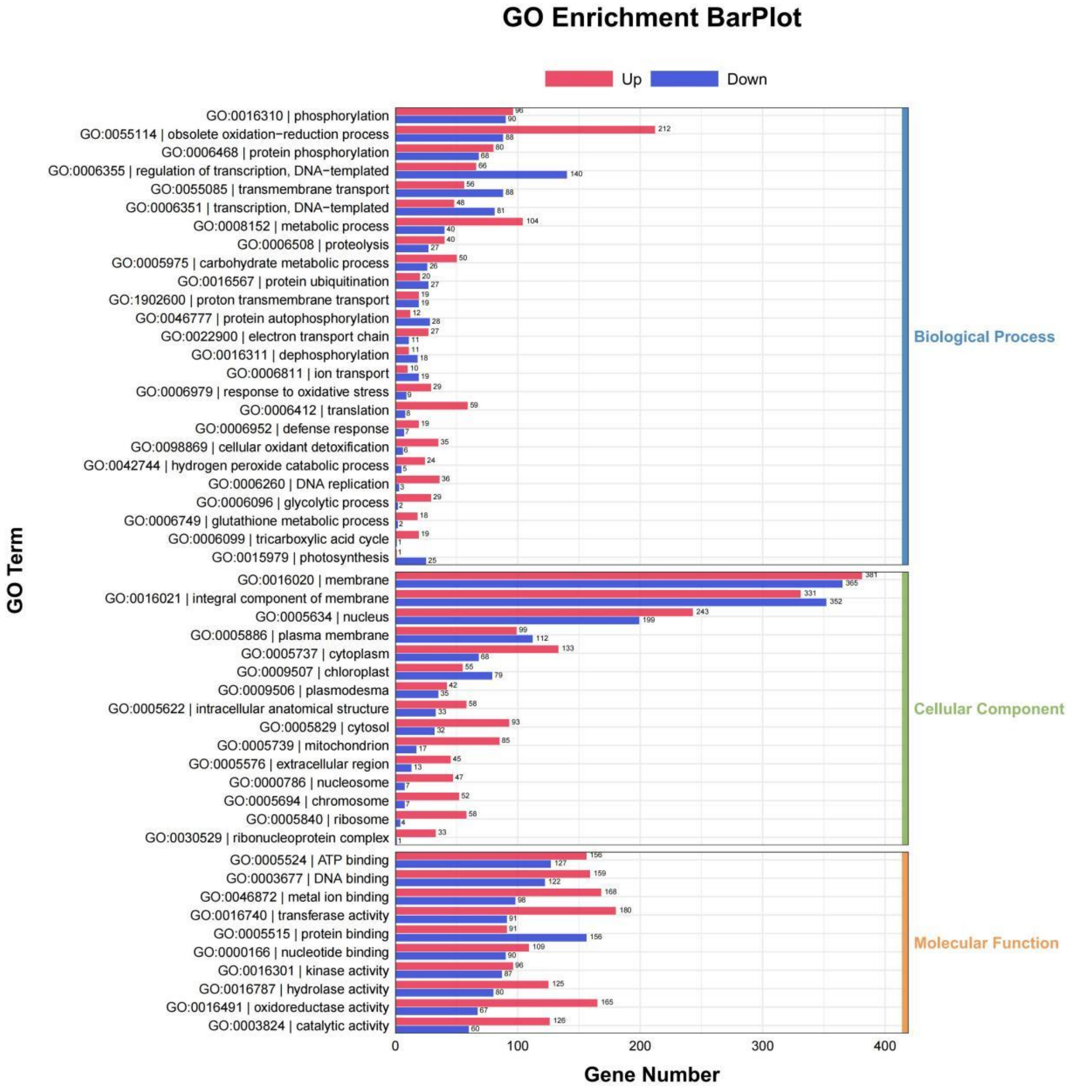


**Figure S1.** Histogram of GO enrichment analysis of differential genes with the same expression trend in ZC17 and CH72. The red and blue bars indicate the number of up- and down-regulated DEGs, respectively.

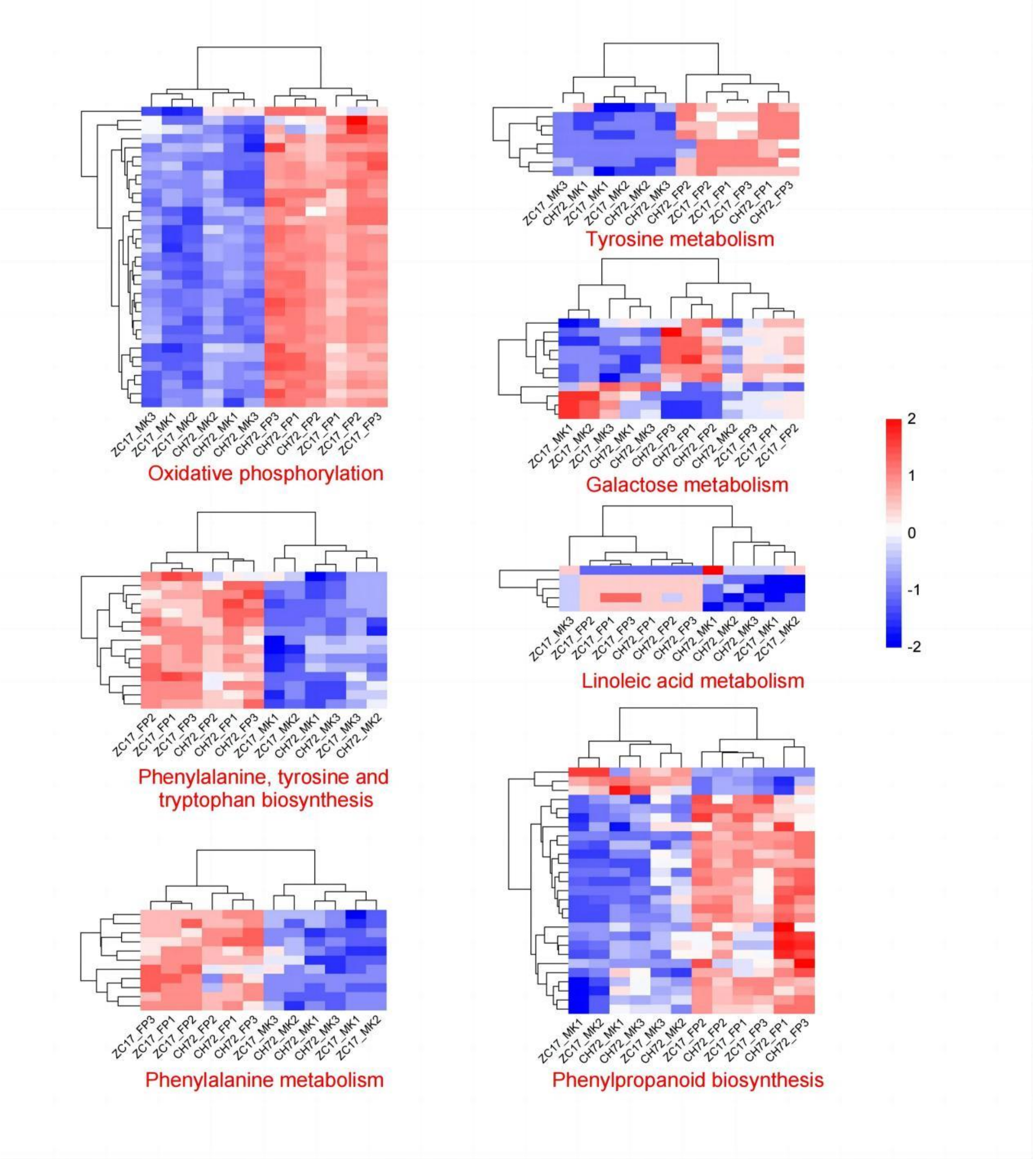


**Figure S2.** Heatmap of expression clustering of DEGs in pathways closely related to plant disease resistance.

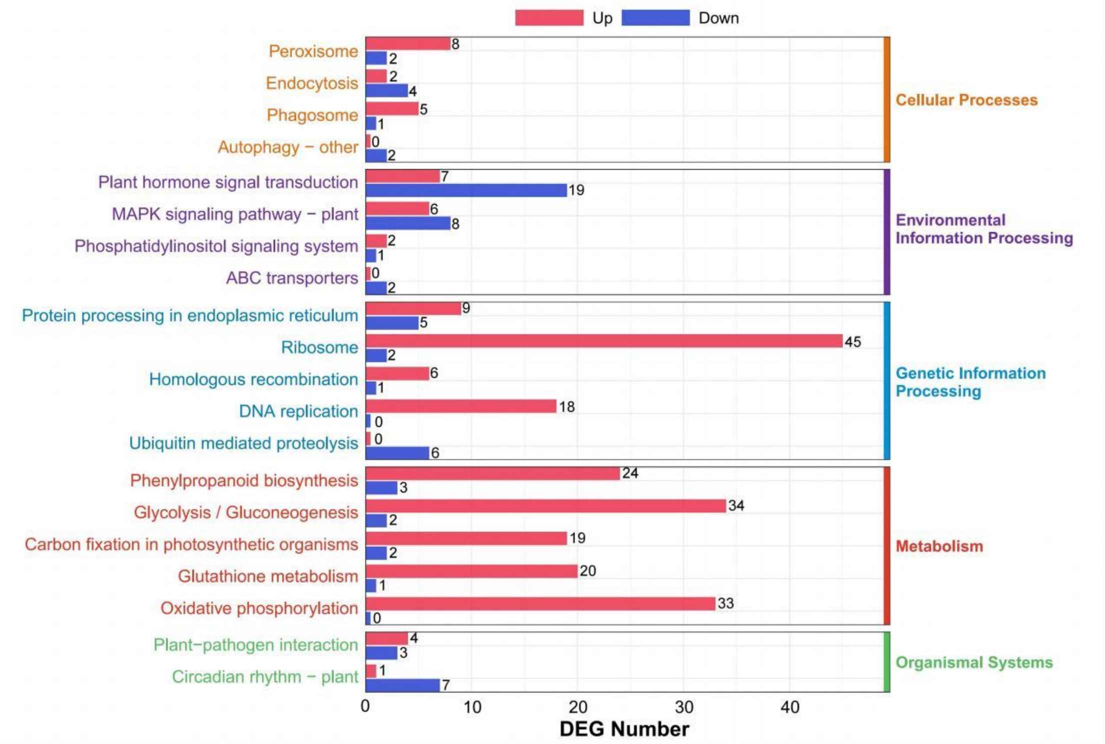


**Figure S3.** Histogram of KEGG enrichment analysis of differential genes with the same expression trend in ZC17 and CH72. The red and blue bars indicate the number of up- and down-regulated DEGs, respectively.

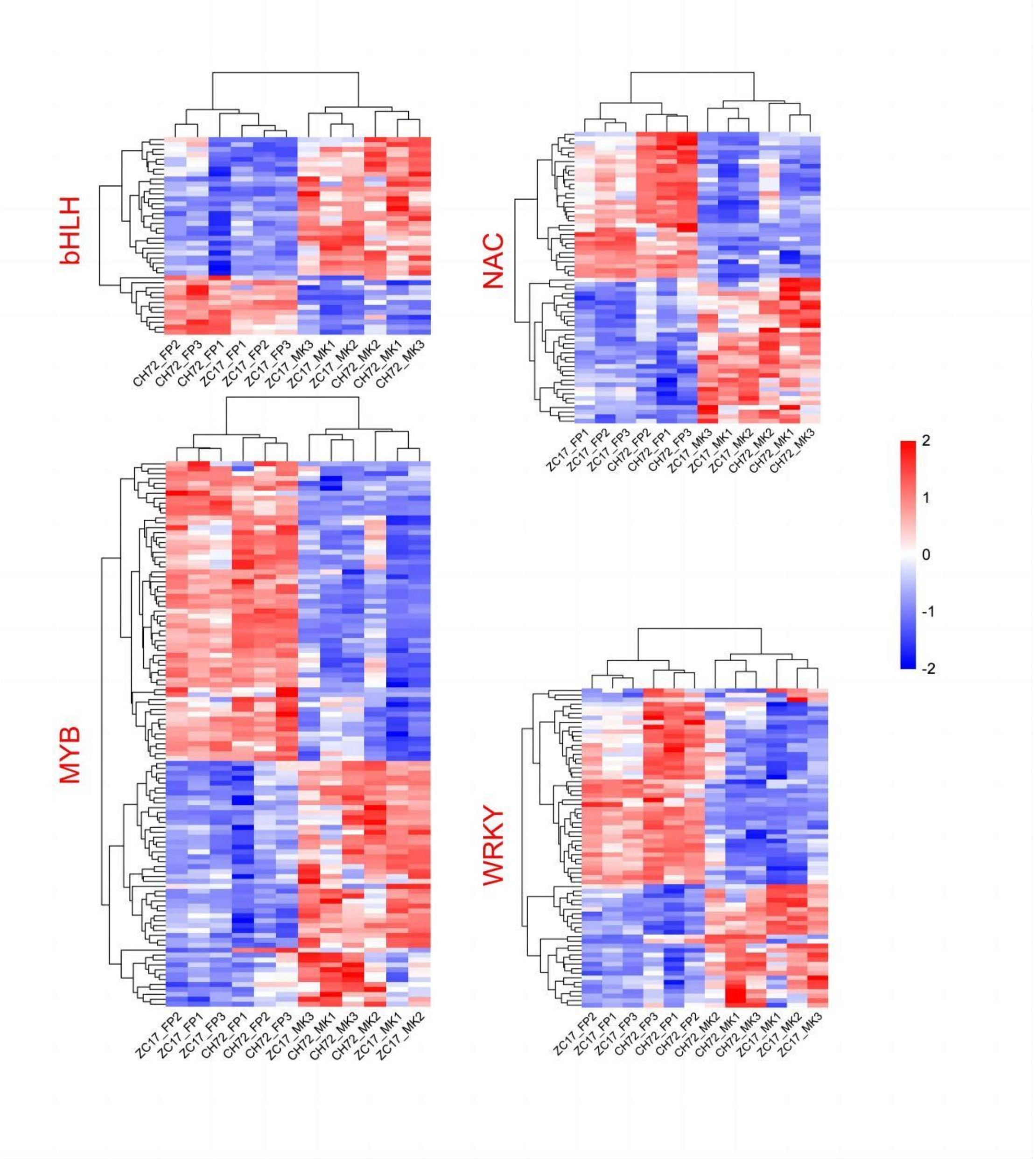


**Figure S4.** Heatmap of expression clustering of transcription factor families closely related to plant disease resistance.
